# Exploring the Role of Levonorgestrel Intrauterine System (LNG-IUS) as a Method of Emergency Contraception (EC)

**DOI:** 10.7759/cureus.31959

**Published:** 2022-11-28

**Authors:** Snigdha Kumari, Avir Sarkar, Anshul Kulshreshtha, Rinchen Zangmo, K K Roy

**Affiliations:** 1 Obstetrics and Gynecology, Post Graduate Institute of Medical Education and Research, Chandigarh, IND; 2 Obstetrics and Gynecology, All India Institute of Medical Sciences, New Delhi, IND; 3 Obstetrics and Gynecology, All India Institute of Medical Sciences, Delhi, IND

**Keywords:** unmet need for contraception, contraceptive pills, postcoital contraception, emergency contraception, levonorgestrel intrauterine system

## Abstract

Copper T 380-A (CuT380A) intrauterine device (IUD) has been an effective method of emergency contraception (EC). Levonorgestrel intrauterine system (LNG-IUS) has not been approved by the Food and Drug Association for EC till now. There are few studies that provide data regarding the efficacy of LNG-IUS as EC. This systematic review tried to explore the efficacy of LNG-IUS in preventing accidental pregnancies up to five days of unprotected intercourse.

A systematic review of the published studies on the use of LNG-IUS as EC was done. All randomized trials, prospective cohorts, retrospective cohorts. and case-control study designs pertaining to this topic were included in this review. Abstracts were retrieved and reviewed by two authors independently. Variables pertaining to socio-demographic parameters, EC use-related variables (reason for use, frequency, time elapsed since coitus), and those associated with sexual habits were selected and recorded.

A total of six articles were rendered for the review. High school students and those attending college accounted for 36.8%-51.8% of the study population. Data on the reason for seeking EC showed noncompliance to routine contraception as the most common reason, followed by failure of withdrawal method, breach of barrier contraception, and unplanned intercourse. With a negligible failure rate, LNG-IUS seemed to be a good alternative to the existing copper EC.

Considering the plethora of noncontraceptive benefits associated, LNG-IUS can be safely provided as an option of EC in the cafeteria approach within five days of unprotected intercourse.

## Introduction and background

Although a number of methods of emergency contraception (EC) are in use worldwide, the United States-Food and Drug Administration (US-FDA) has approved only two methods to date: oral levonorgestrel (LNG) also referred to as Plan B and oral ulipristal acetate. Copper T 380A (CuT380A) intrauterine device (IUD) has also been an effective method of EC with an almost negligible failure rate of less than 0.1% [1]. Though not approved by the FDA, it is being widely used as EC for up to 120 h of unprotected intercourse [1]. Throughout the history of medicine, levonorgestrel intrauterine system (LNG-IUS) or Mirena 52 mg has not been in the recommendation as a method of EC, the primary reason being the nonavailability of data to support its role in the above-mentioned field of contraception.

Recent studies have shown that women prefer LNG-IUS to copper IUD for long-term reversible contraception [2]. It could be probably because of the additional benefits of reduction in blood loss during cycles (thereby preventing anemia) and a significant decrease in dysmenorrhoea associated with its use. However, practice recommendations still advise women to follow the age-old methods of oral EC in cases of unprotected intercourse [3]. This has forced researchers to explore the role of LNG-IUS as a method of EC. Today, there are only a handful number of studies that provide some data regarding the efficacy of LNG-IUS as EC. Due to a lack of an ample number of researches, the FDA has not approved its role. Through this review, we tried to summarize all existing studies exploring the efficacy of LNG-IUS in preventing accidental pregnancies up to five days of unprotected intercourse.

## Review

The aim of this review was to find out the role of LNG-IUD in preventing unplanned pregnancies through a comprehensive search of the literature.

The PRISMA 2020 statement was followed for conducting and reporting this review [4]. We performed a systematic review of the literature published on the use of LNG-IUS as EC with the aim to discern its role in preventing unplanned pregnancies. The results of this comprehensive review would help a woman to select an emergency contraceptive method that best suits both her immediate and future needs. The review was performed in the following steps: a) determining the research question, b) literature search to identify relevant published studies, c) selecting the studies appropriate for recruitment in the review, d) classifying and summarizing the data in a tabular form, and e) reporting the relevant results.

**Figure 1:**
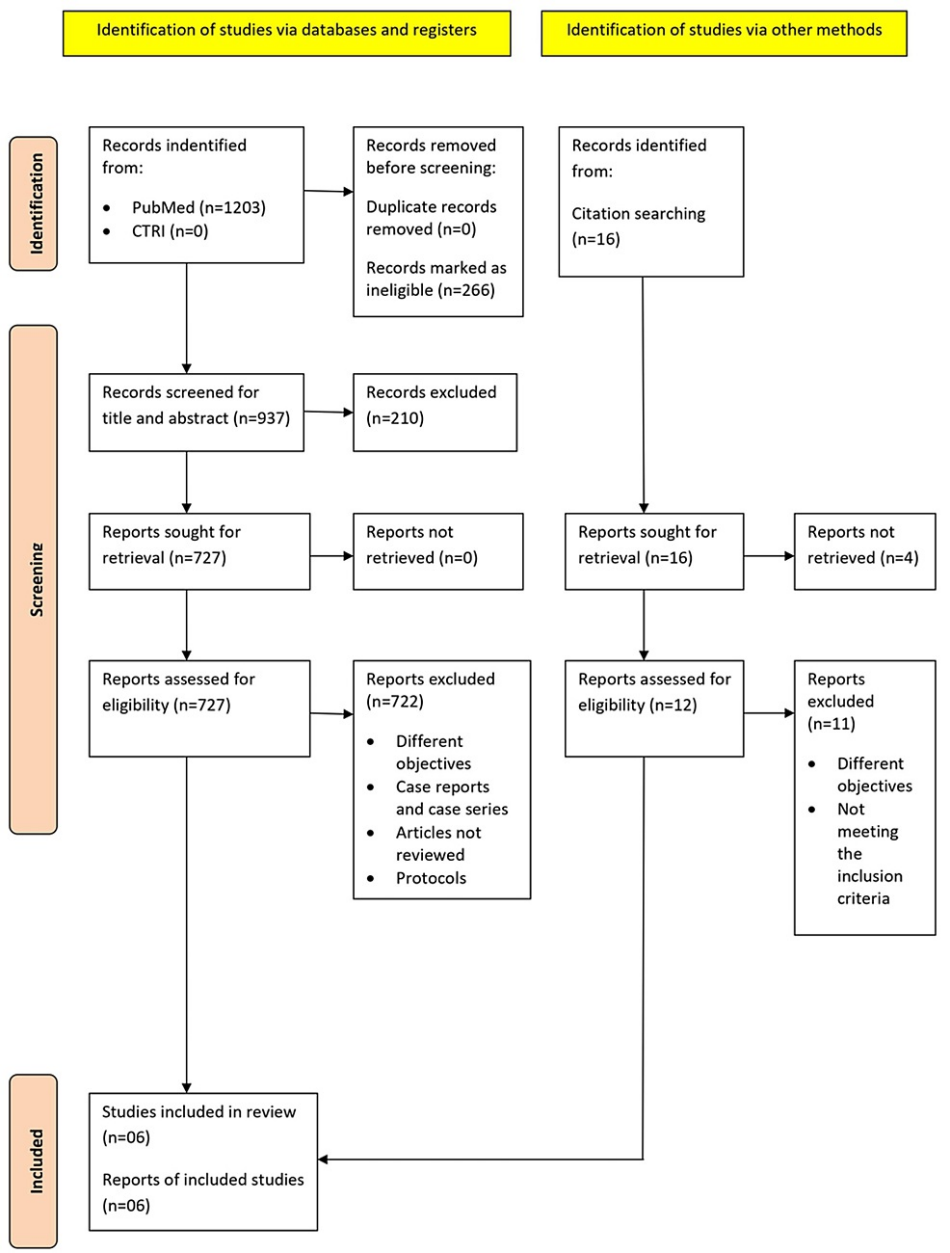
PRISMA 2020 flowchart followed for conducting and reporting this review. PRISMA, Preferred Reporting Items for Systematic Reviews and Meta-analyses

(a) *Determining the research question*: We designed the research question as - whether LNG-IUS is suitable to be used as an emergency contraceptive.

(b) *Search strategy*: A comprehensive literature and bibliographic search were carried out to identify studies that assessed pregnancy risk after LNG-IUS placement. A pre-defined search strategy was followed using a combination of keywords: ((levonorgestrel) OR (intrauterine device)) AND (((post coital) OR (emergency)) AND (contraception)) published on PubMed and Clinical Trial registry bodies [e.g., Clinical Trial Registry of India (CTRI)] since inception till August 31, 2022. The full texts of relevant manuscripts were obtained to assess and analyze them independently through a simple grid that was designed for this study. We screened all published articles evaluating the efficacy of LNG-IUS as a method of EC. For articles not captured by the electronic search, we extracted data by doing a manual search using the references in the original articles. PRISMA 2020 statement was used for including the relevant studies for the review (Figure 1).

(c) *Inclusion criteria*: We wanted to evaluate the efficacy of LNG-IUS in preventing unwanted pregnancies. Hence published articles related to this research topic were included in this review.

- All randomized or quasi-randomized controlled trials,

- Prospective cohort, retrospective cohort, and case-control study designs pertaining to this topic were included in this review.

- Articles that reported the contraceptive role of LNG-IUS as primary or secondary outcomes were recruited in this review.

(d) *Exclusion criteria*: Articles with at least any one of the following criteria were excluded from this review:

- articles published in a language other than English,

- study protocols or trials without results,

- articles in pre-print (not yet peer reviewed),

- case reports, case series and cross-sectional studies, and

- studies where the role of LNG-IUS for controlling abnormal uterine bleeding has been explored. 

(e) *Selecting studies appropriate for recruitment in the review*: To identify which studies were to be included in this review, the following criteria were used. Studies with the sample population being women attending family planning clinics seeking EC were eligible for recruitment. The intervention was to determine the efficacy of the LNG-IUS as an EC and to support its use in women who would like to use a highly effective reversible method in the same setting for future contraceptive care. The outcome was the assessment of pregnancy status defined as a positive urine pregnancy test conducted in the clinic or at home after one month of IUD placement. Abstracts were retrieved and reviewed by two authors independently (authors AS and SK). To gain the final decision on inclusion or exclusion, the full-text manuscripts of the studies fulfilling the selection criteria were reviewed in detail. Any disagreements with regard to study eligibility were resolved by discussion and consensus with the third and fourth authors (AK and RZ).

(f) *Data extraction*: Information was collected on the aim of the study, study design, sample size, participants, variables related to the use of EC, results, and conclusion. The gathered variables were socio-demographic variables (age, ethnicity, relationship status, income), EC use-related variables (reason for use, frequency, time elapsed since coitus), variables associated with sexual habits (obstetrics history/parity, intrauterine infection within past three months), and the method of EC selected were recorded. The studies were evaluated and assessed to ensure that the minimum quality standards were met.

(g) *Quality assessment*: Methodological quality was as per Cochrane Systematic Review Guidelines and GRADE-PRO approach was used for rating the quality of a body of evidence. The individual studies were evaluated separately by AS and SK and in case of a dilemma regarding the quality assessment, it was resolved after a detailed discussion with AK and RZ.

(h) *Reporting the relevant results and summarizing the data in a tabular form:* After the selection of the relevant studies, the obtained data were tabulated as shown in Table 1. The relevant data were summarized and reported. 

**Table 1:** Comparative data of the studies included in the review. IUD, intra-uterine device; EC, emergency contraception; LNG-IUS, levonorgestrel intrauterine system; UPT, urine pregnancy test

Type of study	Year of publication	Authors	Duration of study	Objectives of the study	Study population	Outcome	Inference
Retrospective study	2014	Prine et al. [5]	5.5 years	Retrospective chart review to assess the efficacy of LNG-IUS as a method of emergency contraception	Two groups of women where LNG-IUS insertion was done at high-risk time in their menstrual cycle: 1. who admitted to unprotected intercourse and got additional Plan B (88 patients) 2. who did not have unprotected intercourse (499 patients)	In Group 1, two pregnancies occurred, one had LNG inserted on Day 21 of her cycle and received Plan B one day prior to insertion. She had multiple unprotected sex and so pregnancy was not a result of LNG-IUS failure. Other was an LNG failure where pregnancy occurred 3 months after insertion. No pregnancy occurred in Group 2.	There may be mechanism of action whereby LNG-IUS works as EC
Prospective cohort study	2016	Turok et al. [6]	16 months (June 2013 to September 2014)	1. Assessment of IUD preference among women desiring EC 2. Role of concurrent oral LNG plus LNG IUS as EC	All women desiring EC during the study period were asked to choose between CuT380A IUD or (concurrent oral LNG plus LNG IUS) Out of 188 participants, 67 (36%) selected CuT380A and 121 (64%) selected Oral LNG + LNG IUS Out of 188 women, 10 had failed insertion, 1 had uterine didelphys and 1 patient withdrew So, finally, 176 women received EC	1. Study participants preferentially chose oral LNG + LNG IUS as the desired method more than CuT380A. 2. The probability of pregnancy after 2 weeks was checked by UPT. No pregnancy was noted in the group seeking CuT380A. The only pregnancy noted in the LNG group occurred in a woman who was non-compliant and had multiple episodes of unprotected intercourse more than 5 days prior to EC.	Neither group had EC failures. Including the option of concomitant LNG-IUS with oral LNG may increase compliance to EC
Prospective cohort study	2017	Sanders et al. [7]	1 year	Assessment of 1 year continuation rate of CuT380A and LNG IUS among the women who chose EC in Study 6	Out of 176 participants, 98 (67%) had IUD in situ after 1 year of follow-up.	Continuation rates did not differ by IUD type (60% for CuT380A and 70% for LNG IUS, who had initially received oral LNG)	1. Providing IUD option for EC increases compliance 2. LNG-IUS seems to be a good option for EC with negligible failure rate
Non-inferiority randomized controlled trial	2021 Jan	Turok et al. [8]	41 months (August 2016 to December 2019)	Role of LNG-IUS alone as a method of EC and comparing its efficacy with CuT380A	Among 355 participants randomly assigned to LNG-IUS and 356 to CuT380A groups, 317 and 321, respectively, received interventions and provided 1 month UPT results.	1. The primary outcome was pregnancy confirmed by a positive UPT after 1 month of EC- IUD insertion 2. Out of 317 participants in the LNG IUS group, 1 had pregnancy (0.3% failure). There was no pregnancy among 321 participants in CuT380A group (0% failure).	LNG-IUS was non-inferior to CuT380A for EC
Secondary analysis of randomized control trial (Study 8)	2021 Jun	Fay et al. [9]	41 months (August 2016 to December 2019)	1. Assessment of frequency of use of backup contraception after IUD placement as EC 2. Pregnancy rates 1-month after IUD placement (LNG-IUS or CuT380A) as EC	518 women with unprotected intercourse in the preceding 5 days were randomly allocated to either LNG-IUS or CuT380A as a method of EC. Pregnancy status was checked 1 month later with UPT.	1. Rapid return to sexual activity was common after EC and backup contraception was rarely used after EC (16.4%) 2. No pregnancies occurred among women using LNG-IUS (0/138) and CuT380A (0/148) as EC	No pregnancy was noted after placement of an IUD for EC, both for LNG-IUS and CuT380A, even in those who do not use backup contraception
Secondary analysis of randomized control trial (Study 8)	2021 Jul	BakenRa et al. [10]	41 months (August 2016 to December 2019)	Assessment of pregnancy risk after IUD placement (as EC) by number and timing of unprotected intercourse episodes in the prior 14 days	Study participants were randomly allocated to LNG-IUS and CuT380A groups in Study 7	Pregnancy risk difference did neither significantly differ by single vs multiple unprotected intercourse, nor by intercourse less than 5 days vs 6-14 days before IUD placement	1 month pregnancy risk remains low, regardless of frequency or timing of unprotected intercourse after both LNG-IUS and CuT380A as EC

(i)* Results of the study characteristics*: The initial search ((levonorgestrel) OR (intrauterine device)) AND (((post coital) OR (emergency)) AND (contraception)) identified a total of 1203 studies via searching through database and clinical registry and 16 studies via manual searching of citations. The database search was carried out as follows:

1. Levonorgestrel (n=6647)

2. Intrauterine device (n=15,093)

3. #1 OR #2 (n=19,461)

4. Emergency contraception (n=4086)

5. Post coital contraception (n=300)

6. #4 OR #5 (n=4190)

7. #3 AND #6 (n=635)

8. ((post coital) OR (emergency)) AND (contraception) (n=6325)

9. #3 AND #8 (n=1203)

After screening the title and abstract of all the retrieved articles, 937 studies were included. After excluding the nonrelevant studies, a total of 739 studies (727 through databases and 12 through manual retrieval) were assessed for eligibility. A careful analysis of the eligible text resulted in six articles being rendered out for review (Figure 1).

(j)* Description of the included studies*: The selected studies were carried out between 2013 and 2021 (Table 1). One retrospective study was conducted in Israel [5] and two prospective cohort studies [6-7] were conducted in the United States. A noninferiority randomized controlled trial from the United States [8] along with its secondary analyses [9-10] were the major studies included in this review. The data in these studies were collected from emergency services with a focus on women who requested EC. The ages of women enrolled across the selected studies ranged from 18 to 35 years. The follow-up period differed from 1 year to 5.5 years. The details of the study objectives, study population, interventions, study outcome, and data assessment reporting are shown in Table 1.

(k) *Methodological quality of studies*: Review authors assessed the quality of the included studies with the aid of the JBI critical appraisal checklist. The quality assessment of the randomized controlled trials and cohort studies were done separately as shown in Tables 2-3.

**Table 2:** Quality assessment of the RCTs included in the review using JBI critical appraisal checklist. RCTs, randomized controlled trials

Questions	Turok et al. [8]	Fay et al. [9]	BakenRa et al. [10]
1. Was true randomization used for assignment of participants to treatment groups?	Yes	Yes	Yes
2. Was allocation to treatment groups concealed?	Yes	Yes	Yes
3. Were treatment groups similar at the baseline?	Yes	Yes	Yes
4. Were participants blind to treatment assignment?	Yes	Yes	Yes
5. Were those delivering treatment blind to treatment assignment?	Yes	Yes	Yes
6. Were outcome assessors blind to treatment assignment?	Yes	Yes	Yes
7. Were treatment groups treated identically other than the intervention of interest?	Yes	Yes	Yes
8. Was follow up complete and if not, were differences between groups in terms of their follow up adequately described and analyzed?	Yes	Yes	Yes
9. Were participants analyzed in the groups to which they were randomized?	Yes	Yes	Yes
10. Were outcomes measured in the same way for treatment groups?	Yes	Yes	Yes
11. Were outcomes measured in a reliable way?	Yes	Yes	Yes
12. Was appropriate statistical analysis used?	Yes	Yes	Yes
13. Was the trial design appropriate, and any deviations from the standard RCT design accounted for in the conduct and analysis of the trial?	Yes	Yes	Yes

**Table 3:** Quality assessment of the cohort studies included in the review using JBI critical appraisal checklist. JBI, Joanna Briggs Institute

Questions	Prine et al. [5]	Turok et al. [6]	Sanders et al. [7]
1. Were the two groups similar and recruited from the same population?	Unclear	Yes	Yes
2. Were the exposures measured similarly to assign people to both exposed and unexposed groups?	Unclear	Yes	Yes
3. Was the exposure measured in a valid and reliable way?	Unclear	Yes	Yes
4. Were confounding factors identified?	No	Yes	Yes
5. Were strategies to deal with confounding factors stated?	No	Yes	Yes
6. Were the groups free of the outcomes at the start of the study?	Unclear	Yes	Yes
7. Were the outcomes measured in a valid and reliable way?	No	Yes	Yes
8. Was the follow up time reported and sufficient to be long enough for outcome to occur?	No	Unclear	Yes
9. Was follow up complete?	No	Unclear	Yes
10. Were strategies to address incomplete follow up utilized?	No	No	Unclear
11. Was appropriate statistical analysis used?	Yes	Yes	Yes

During the quality assessment of the randomized controlled trials, a study by Turok et al. [8] was found to have a low risk of selection bias as proper block randomization was used in the trial design, and the blinded sequences were uploaded in REDCap prior to the recruitment of participants. Similarly, the risk of attrition, detection, and reporting bias was also rated as low since the outcome data was complete and there was no selective reporting of the results. Studies by Fay et al. [9] and BakenRa et al. [10] were secondary analyses of the randomization performed by Turok et al. and were also rated to have a low risk of bias (Table 2). 

**Figure 2:**
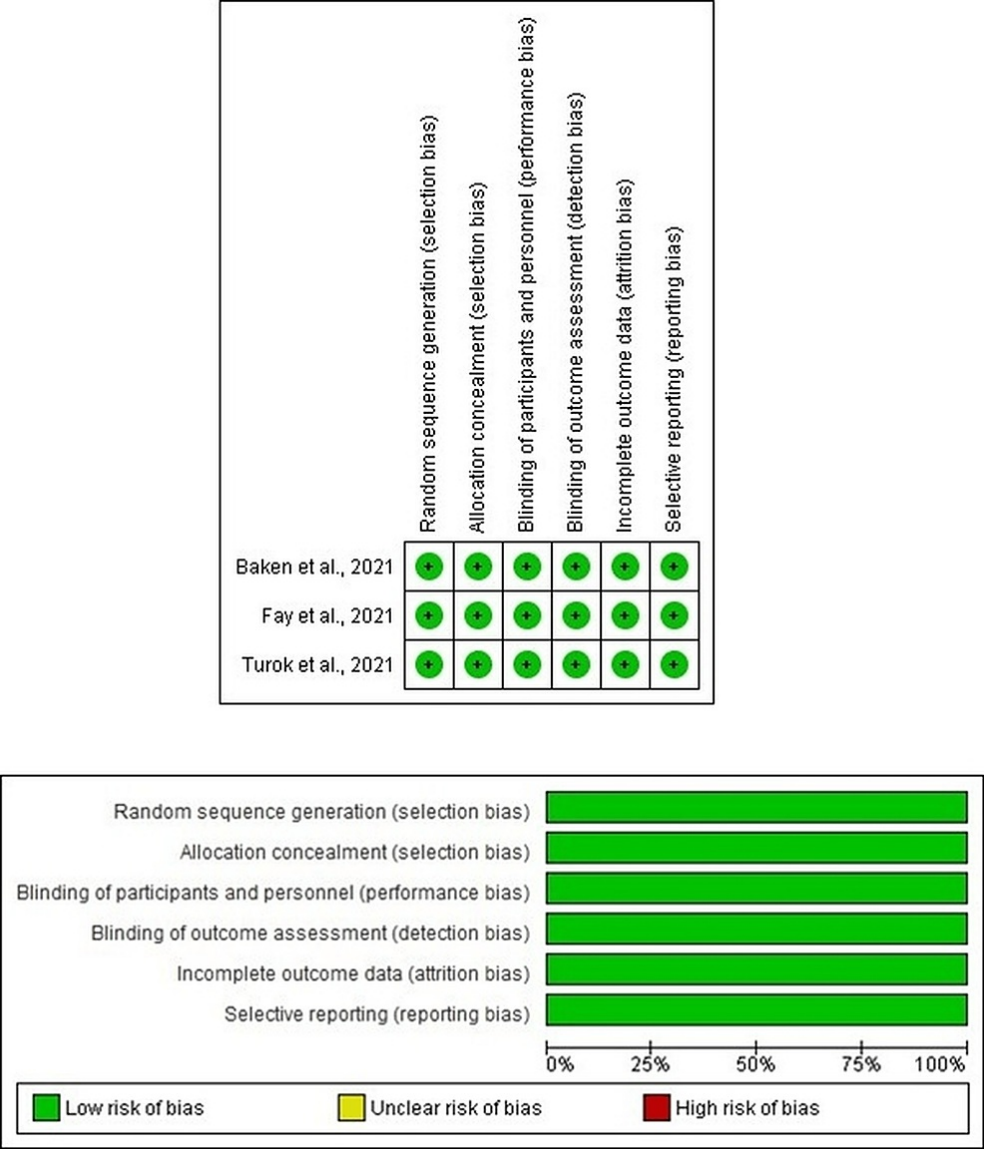
Quality assessment of included RCTs using RevMan 5.4 plots. RCTs, randomized controlled trials

**Figure 3:**
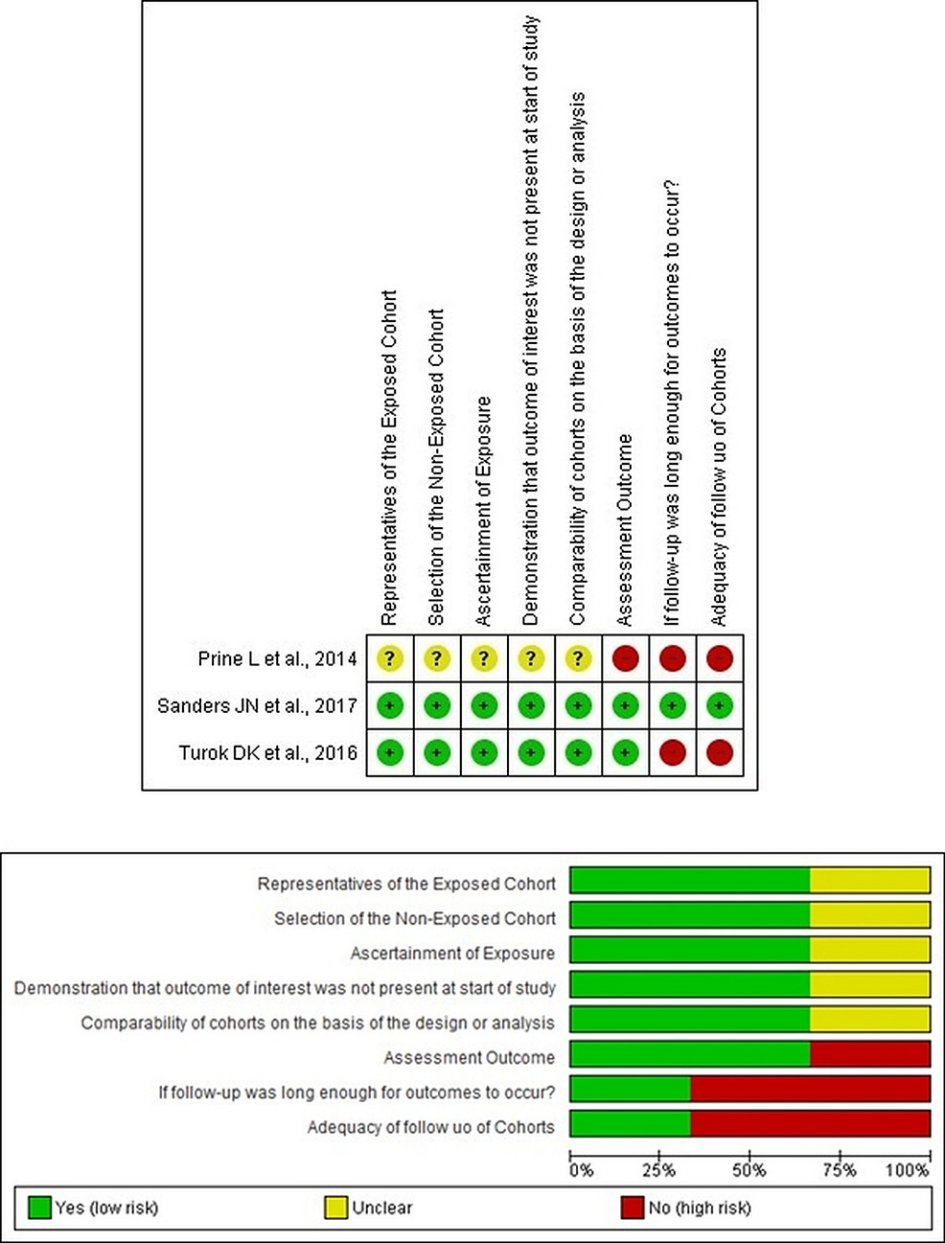
Quality assessment of included cohort studies using RevMan 5.4 plots.

On analyzing the retrospective and prospective cohort studies included in this review, a study by Prine et al. [5] had unclear data regarding the ascertainment of exposures, selection of the nonexposed cohort, and representativeness of the exposed cohort. Moreover, data pertaining to the comparability of cohorts on the basis of design and analysis was lacking in the study. There was no prospective follow-up of the observed cohort. Hence, the risk of bias ranged from unclear to high. Studies by Turok et al. [6] and Sanders et al. [7] had true representativeness of the exposed and nonexposed cohorts and demonstrated comparability between the groups in terms of designs and analyses. Long-term follow-up of the recruited subjects was not done by Turok et al. [6] and was hence found to have a high risk of bias in terms of follow-up. However, a one-year prospective follow-up of their recruited cohort was completed by Sanders et al. [7] and was rated to have a low risk of bias in terms of follow-up of cohorts. Hence, the risk of bias varied widely ranging from low to high based on the respective quality assessment question (Table 3). The methodological quality of the studies has been depicted graphically using the RevMan 5.4 plots (Figure 2 for randomized controlled trials and Figure 3 for cohort studies). 

(l) *Discussion*: The demand for EC confirms a tremendous unmet public health need for more reliable and more effective contraception [8]. The availability of a greater number of contraceptive options is associated with increased satisfaction with and continuation of the use of the method and decreased unintended pregnancy [11]. Unfortunately, the wide availability of oral EC pills has not reduced abortion rates over the years [12-13]. Providing non-users and users of less effective methods of contraception with highly effective ones is an efficient strategy to reduce rates of unplanned pregnancy and abortion. Application of this principle has reduced repeat abortions for women who receive immediate post-abortal IUD insertion [14].

The efficacy of EC depends on a number of factors like age, body mass index (BMI), the number of unprotected intercourse, the presence of sexual intercourse in the fertile window, and the timing of EC use after intercourse. Intrauterine EC can be a better alternative to oral EC. It is associated with a lesser failure rate, provides long-term ongoing contraception, and is not associated with nausea and vomiting. Its efficacy is not affected by BMI. Laboratory data support the potential for LNG to directly interfere with sperm transport, sperm capacitation, acrosome reaction, and oviduct transport [15]. Although there is negligible risk of abdominal cramps and bleeding during insertion, LNG IUS has additional benefits of reducing blood loss and dysmenorrhoea, thereby improving the hematological parameters. After being explained about the risks and benefits of copper T versus oral LNG and LNG IUS majority of women chose LNG as the preferred method for EC {121 (64%) in the LNG arm vs 61 (36%) in the copper T arm} [6].

(m)* Socio-demographic variables of the study subjects*: In five of the six articles the majority of women who sought EC were reported to be aged between 18 and 35 years. Among women requesting EC, most were either single and actively dating or separated and divorced. Regarding employment, five studies collected data on this variable. All five studies showed that the largest percentage of the population under study was students. High school students and those attending college accounted for 36.8%-51.8% of the study populations in these studies. Information provided on BMI showed that subjects with overweight accounted for between 21.4% and 41%. The study population with obesity ranged from 16% to 27.6%, placing them at higher risk for contraception failure.

(n) *EC use-related variables*: Data on the reason for seeking EC in the study population showed no contraception usage as the most common reason (ranging from 40.7% to 52%), followed by contraceptive failure. LNG-IUS users who continued using their device at 12 months was 70% as per one of the studies [7]. Out of them, 71% of women continuing categorized their experience as very satisfied. The most common reason for discontinuation is pain and bleeding [7-8].

With reference to the user profile, in the four studies that included this variable, it showed that a very high percentage of women seek EC within five days of unprotected coitus. Only a small proportion of participants reported unprotected intercourse beyond five days before IUD placement extending till an elapse of 14 days before reporting [6, 8, 10]. Common reasons for EC use included nonusage of any of the established methods of contraception, failure of withdrawal method of contraception, breach of barrier contraception, missed a dose of contraception, and unplanned intercourse. With respect to articles elucidating on the frequency of episodes of unprotected intercourse, it appeared that the majority of participants stated a single episode of unprotected coitus before seeking EC use than those reporting multiple episodes [6, 10]. Some 43.7% of women had a history of multiple episodes of unprotected intercourse and 14% had at least one episode of unprotected intercourse more than five days before IUD placement [10]. It is noteworthy that the majority of candidates resumed intercourse within one month of IUD placement. In the same study, 66.4% of participating women commenced engaging in unprotected intercourse during the same menstrual cycle, more so, within seven days of IUD placement.

(o) *Sexual habits associated variables*: In a few studies, obstetrics history was recorded, reporting that 55% of women surveyed have had a pregnancy in past [6-7]. Data on the sexually transmitted infection screen was recorded in one study and appeared to be positive in 7% of interviewees. In another study, 36.7% stated to have more than once per week frequency of coitus [9].

(p)* Comparison of LNG-IUS vs copper-T in terms of EC*: In the retrospective chart review by Prine et al., two groups of women were studied where LNG-IUS insertion was done at the high-risk time in their menstrual cycle [5]. One group comprised women who admitted to unprotected intercourse and got additional Plan B and the second group who did not have unprotected intercourse. Overall, IUDs for EC increase compliance among users. With a negligible failure rate, LNG-IUS seemed to be a good alternative to the existing copper intrauterine EC.

In the prospective study comparing the efficacy of concurrent oral LNG and LNG-IUS with copper intrauterine EC, both groups have negligible EC failure rates [6]. However, including the option of concomitant LNG-IUS with oral LNG increased compliance to EC in a one-year prospective follow-up [7]. In the randomized trial by Turok et al, LNG-IUS alone had shown to have a negligible EC failure rate like copper-T 380-A (0.3% vs 0%) [8]. No pregnancy was noted in both arms even in those who did not use any method of backup contraception [9]. Moreover, failure rates remain negligible in both arms regardless of the frequency or timing of unprotected intercourse [10].

(q) *Strengths*: The present study reviewed the existing literature to explore the efficacy of LNG-IUS as a method of EC. Currently, the use of LNG-IUS as EC is still a debatable topic among clinicians due to the lack of adequate data on its efficacy for this purpose. This review is the first to summarize the results of the available studies till now explaining the increased preferability and efficacy of LNG-IUS as a method of EC. Conclusions from the randomized trial and its secondary analyses will certainly trigger gynecologists to counsel couples around the globe to choose LNG-IUS for EC and its long-term continuation [8-10]. 

(r) *Limitations*: The trials available till now included women in the 18-49 years (reproductive age group). Such selection criteria may limit its generalization to the population at large [8-10]. Selection bias could also be possible since only seven percent of the patients seeking EC were enrolled in the randomized trial [8]. A meta-analysis of the systematic review was not performed. Large scale multicenter trial is required comparing the efficacy of LNG-IUS with other established methods of EC for it to get FDA approval for the said use.

## Conclusions

Levonorgestrel IUD has proved to be noninferior to copper-T as a method of EC. Considering the variety of noncontraceptive benefits, LNG-IUS can be safely provided as an option of EC in the cafeteria approach within five days of unprotected intercourse. However, large multicentric trials are warranted for the formulation of guidelines and approval of this new IUD for the said use.
